# An Unusual Case of Severe Hypertension Presenting With Leg Weakness, Hypokalemia, and Hyperreninemic Aldosteronism

**DOI:** 10.7759/cureus.50376

**Published:** 2023-12-12

**Authors:** Martina Antonello, Sabrina Dalla Mora, Leonardo A Sechi, Andrea Da Porto

**Affiliations:** 1 Department of Medical Area, University of Udine, Udine, ITA

**Keywords:** iperrenicemic aldosteronism, severe hypertension, renovascular hypertension, renal stenosis, takayasu's arteritis

## Abstract

Takayasu's arteritis is a rare vasculitis characterized by granulomatous inflammation of the large vessels, typically occurring in the second or third decade of life and preferentially affecting females. It commonly involves large vessels such as the aorta and its major branches (carotid and iliac arteries). Visceral arterial involvement is uncommon and reported in only a minority of patients. Clinical manifestations of Takayasu arteritis are heterogeneous and could include nonspecific symptoms such as fever of unknown origin, asthenia, myalgias, intermittent claudication, angina, and mild arterial hypertension. The rarity of this disease and the extreme heterogeneity of clinical manifestations often lead to delays in diagnosis, lasting more than three years in some patients. Improving knowledge of its diagnostic workup could help clinicians in prompt clinical suspicion and early diagnosis. Here, we aim to describe a particular case of a 40-year-old woman with severe hypertension symptomatic for dizziness, gait instability, leg weakness, and diffuse cramps caused by renovascular hypertension as the first clinical manifestation of Takayasu's arteritis involving the right renal artery.

## Introduction

Takayasu's arteritis, or "pulseless disease", is a rare vasculitis characterized by granulomatous inflammation of the large vessels. It typically occurs in the second or third decade of life, mostly in females. The rarity of this disease and the extreme heterogeneity of clinical manifestations often lead to delays in diagnosis, lasting more than three years in 20% of patients [1]. To assist clinicians in diagnosing Takayasu's arteritis, the American College of Rheumatology released specific diagnostic criteria in 1990, which have been recently reviewed by the European Alliance of Associations for Rheumatology (EULAR) [2]. The major clinical findings considered for suspecting Takayasu's arteritis include ≤60 years of age at diagnosis and evidence of inflammation in the aorta or its branch arteries, confirmed by specific vascular imaging. Additional and minor clinical criteria that could be included for diagnosis are female sex, angina or ischemic cardiac pain, arm or leg claudication, vascular bruit of a large artery, reduced pulse in the upper extremity, and reduction or absence of the pulse of the carotid artery or tenderness of the carotid artery. Takayasu's arteritis commonly affects large vessels such as the aorta and its major branches (carotid and iliac arteries). Visceral arterial involvement is uncommon and reported in only a minority of patients. Clinical manifestations of Takayasu's arteritis are heterogeneous and could include many nonspecific symptoms such as fever of unknown origin, asthenia, myalgias, intermittent claudication, angina, and mild arterial hypertension [1]. Although renal arteries are frequently involved by inflammation in Takayasu's arteritis, severe steno-occlusive lesions of the renal artery are rare [3]. To date, only very few cases of severe hypertension due to severe hyperreninemic aldosteronism are reported in current scientific literature as clinical manifestations of Takayasu's arteritis.

## Case presentation

A 40-year-old female patient came to our attention from the emergency room for a clinical study of a severe form of hypertension associated with dizziness, gait instability, and diffuse cramps. Her past medical history included only a recent biopsy for a breast nodule identified as a local lymphocytic vasculitis. She didn't take any drugs or homeopathic products, had no history of allergies, didn't smoke, and had no family history of cardiovascular disease. Her current medical history started six months earlier, during which the patient developed progressive hypertension and started to complain of headaches, weakness in the legs, and diffuse muscular cramps. For this reason, the patient visited her general practitioner many times, and on two occasions, she was sent to the emergency room for extremely high blood pressure values (up to 220/110 mmHg) associated with neurological symptoms (dizziness, headache, gait impairment). On these occasions, clinical and neurological examinations were stated to be unremarkable, and a CT scan of the head showed no abnormalities; therefore, the patient was discharged with advice to monitor blood pressure at home and book a cardiological evaluation.

Once hospitalized in our clinic, during physical examination, we found an abdominal bruit and diminished peripheral (femoral and tibial) pulses bilaterally. Blood pressure (BP) ranged between 200/110 mmHg and 180/100 mmHg during the hospital stay. Other vital signs were normal. A 24-hour continuous monitoring of blood pressure revealed a BP mean value of 183/97 mmHg with a non-dipper profile. The lab tests are summarized in Table 1. The main findings were decreased potassium (which was refractory to supplementation), impaired renal function, and minimal elevation of the erythrocyte sedimentation rate. Further testing has been done to exclude secondary causes of hypertension, revealing a significant increase in renin and aldosterone with a low aldosterone/renin ratio. In light of these findings, we suspected renovascular hypertension.

**Table 1:** Laboratory test for the diagnostic workup of secondary hypertension GOT-AST - glutamic oxaloacetic transaminase - aspartate transaminase; GPT-ALT - glutamic pyruvic transaminase - alanine transaminase; FEIA - fluorescent enzyme immunoassay; anti-CCP - anti-cyclic citrullinated peptide; IIF on Hep-2 - indirect immunofluorescence on human epithelioma type 2 cells; LC-MS/MS - liquid chromatography with tandem mass spectrometry

Blood tests	Results	Reference interval
White blood cells	8.08x10^3/μL	4.00 - 11.00
Red blood cells	4.72x10^6/μL	4.20 - 5.00
Hemoglobin	13.6 g/dL	12.0 - 16.0
Hematocrit	41.2 %	37.0 - 50.0
Mean corpuscular volume (MCV)	87.3 fL	80.0 - 94.0
Platelets	396x10^3/μL	150 - 400
Creatinine	1.2 mg/dL	0.51 - 0.95
Sodium	139 mMol/L	136 - 145
Potassium	2.90 mMol/L	3.5 - 5.10
Magnesium	0.83 mMol/L	0.66 - 1.07
Gamma-glutamyl transferase	15 UI/L	6 - 39
GOT-AST	14 UI/L	4 - 32
GPT-ALT	16 UI/L	4 - 33
Total bilirubin	0.42 mg/dL	0.2 - 1
Direct bilirubin	0.14 mg/dL	0.00 - 0.30
Erythrocyte sedimentation rate	17 mm/h	2 - 15
C-reactive protein	0.88 mg/L	0.00 - 5.00
Procalcitonin	0.06 ng/mL	<10
Cortisol	429 nMol/L	H 8.00: 150.0 - 650.0
Cortisol	245 nMol/L	H 17.00: 70.0 - 350
Adrenocorticotropic hormone (ACTH)	12 pg/mL	5 - 49
Plasminogen tissue activator inhibitor (PAI)	13.4 ng/mL	1.0 - 25.0
Tissue activator inhibitor plasminogen (tPA)	2.0n g/mL	<10
Nocturnal salivary cortisol	1.0 μg/L	0.5 - 2.5
Aldosterone	146.0 ng/dL	4.5 - 28.0
Renin	1203.0 μUI/mL3	3.5 - 46.0
Aldosterone/renin ratio	0.12	<2
Antinuclear antibodies (ANA) screening FEIA	negative	
Anti-citrulline antibodies IgG (anti-CCP - FEIA)	0.7 U/mL	<7 negative
Antibodies anti nucleus (IFI on Hep-2)	absent	
Complement C3	102 mg/dL	90 - 220
Complement C4	37 mg/dL	10 - 40
Anti-treponema pallidum antibodies IgG+IgM	absent	
Urine tests	Results	Reference interval
Aldosterone concentration	60.3 μg/L	
Aldosterone 24h	90.5 μg/24h	1.2 - 28.1
Nor-adrenaline (LC-MS/MS)	95.4 nMol/L	
Nor-adrenaline 24h	315 nMol/24h	90 - 470
Nor-adrenaline/creatinine	38.3 nMol/mMol	
Adrenaline (LC-MS/MS)	13.2 nMol/L	
Adrenaline 24h	44 nMol/24h	0 - 100
Adrenaline/creatinine	5.3 nMol/mMol	
Dopamine (LC-MS/MS)	641 nMol/L	
Dopamine 24h	2115 nMol/24h	400 - 3700
Dopamine/creatinine	257.1 nMol/mMol	

**Figure 1:**
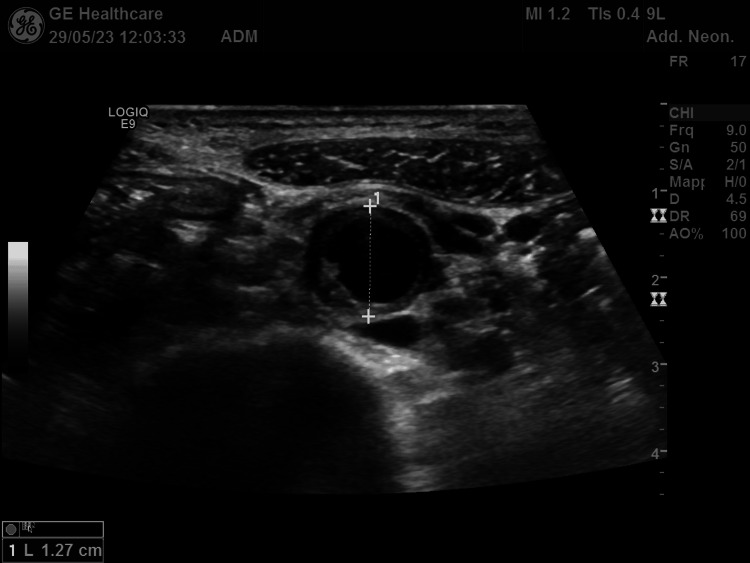
Transversal section of the abdominal aorta with the evidence of diffuse hypoechoic wall thickening

**Figure 2:**
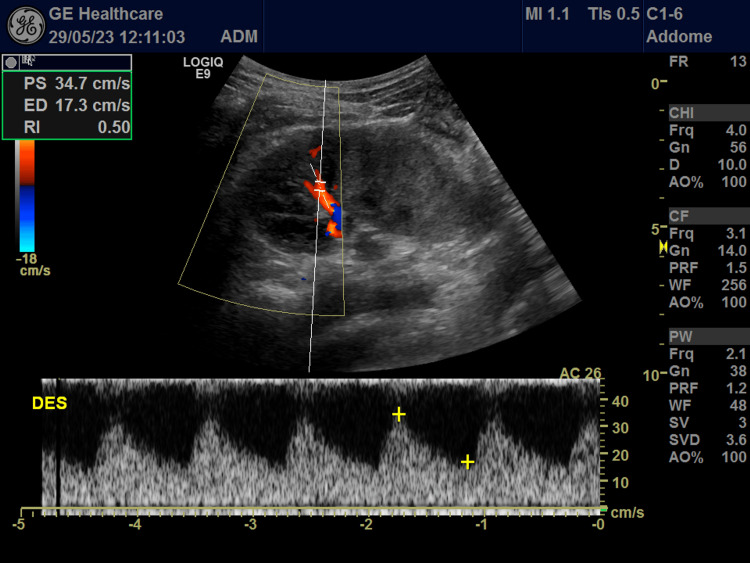
Color Doppler ultrasound of the right renal artery, flowmetric sampling at the level of the intracortical branches Flow paths with reduced resistance index.

**Figure 3:**
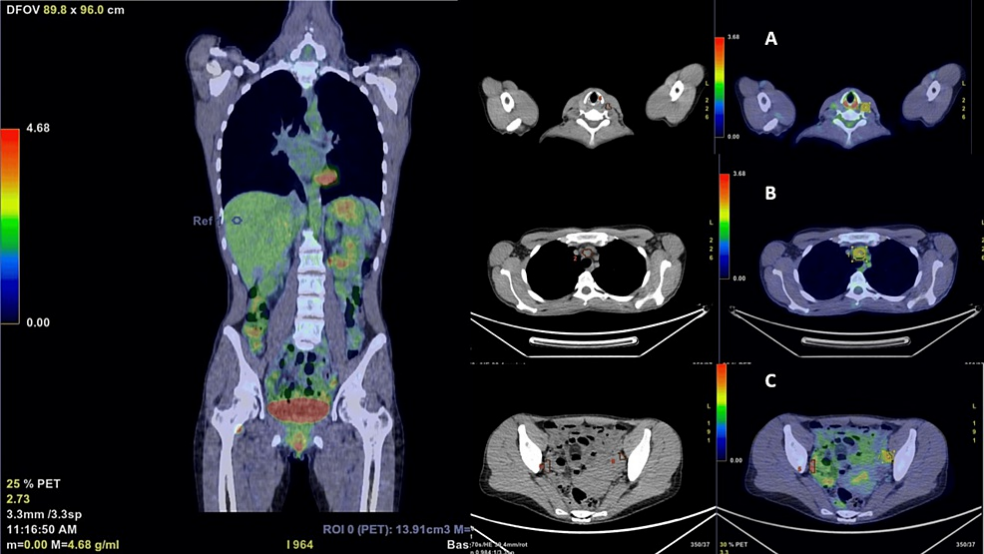
Abnormal concentration of the FDG A: carotid arteries (SUV=3.2); B: aortic arch (SUV=3.2); C: Iliac arteries (SUV=5.6) FDG - fluorodeoxyglucose; SUV - standardized uptake value

Bedside ultrasound examination of the abdomen (Figure 1) showed diffuse wall hypoechoic thickening in the abdominal aorta with complete occlusion of the aorto-iliac bifurcation. Moreover, the right kidney size was decreased (bipolar diameter of 8.7 cm), and the color Doppler ultrasound of the renal arteries showed tight stenosis of the right renal artery with reduced resistance indices sampled in the intrarenal branches compared to the contralateral kidney (Figure 2). The CT angiography confirmed severe stenosis of the right renal artery, showing a complete lack of perfusion of the right common iliac and hypogastric arteries and of the origin of the left iliac axis with downstream reperfusion of the right common and left superficial femoral artery. It also revealed diffuse parietal thickening of the aorta (thoracic and abdominal) and of both internal carotids, raising a suspicion of inflammatory vascular disease. Active inflammatory involvement of arterial vessels was confirmed by the PET-CT scan (Figure 3). The presence of abdominal bruit, leg claudication, and evidence of vasculitis in the aorta and its branches confirmed by vascular imaging, in addition to young age and female sex, allowed us to make a diagnosis of Takayasu's arteritis with a sensitivity and specificity greater than 90% (EULAR's classification score greater than five)[2]. The patient has been treated initially with methylprednisone (1 mg/kg/day) and then with Infliximab. In the meantime, blood pressure and hypokalemia have been reasonably managed with a combination of antihypertensive drugs (amlodipine 10 mg, metoprolol 100 mg bid, chlortalidone 25 mg, spironolactone 100 mg), and potassium chloride (KCL) oral supplements.

## Discussion

Takayasu's arteritis is a rare disease with an incidence of 1.11 cases per million persons a year and predominantly affects women (80-90% of cases) under the age of 40 years [1]. Pathological findings show that in Takayasu's arteritis, granulomatous inflammation typically involves the large vessels. Vascular damage that leads to vascular stenosis is a consequence of cellular infiltration of the middle tunica with local destruction, intimal hyperplasia, and fibrosis. The rarity of this disease and the extreme heterogeneity of clinical manifestations often lead to delays in diagnosis that could last more than three years in 20% of patients [1]. Thus, improving knowledge and diagnostic skills could be useful in clinical practice. Takayasu's arteritis typically affects the subclavian arteries, followed in frequency by the aorta, common carotids, and vertebral arteries. Even though renal arteries could be frequently involved by inflammation, this rarely causes severe local steno-occlusive lesions [3]. For this reason, to date, only very few cases of severe secondary hypertension associated with Takayasu's arteritis are reported in the literature.

Table 2 summarizes the known reports of Takayasu's arteritis associated with severe secondary hypertension. As in our case, the female sex and age at diagnosis of less than 60 years are typical features of the disease [4]; however, severe hypertension due to Takayasu's arteritis has been even reported in the male sex [5,7,9] and in women of older age (65 years) [10]. In most cases, severe hypertension was associated with acute organ damage such as acute kidney injury [4,5,8,11] or neurologic symptoms [4,6,7,9]. Systemic symptoms such as fever or myalgias are also reported [5,6].

**Table 2:** Brief summary of case reports of Takayasu's arteritis associated with secondary hypertension

Author	Case summary
Torun et al. [4]	A 20-year-old female with a remote history of Takayasu's arteritis presented to the emergency department with elevated blood pressure (170/110 mm Hg in the left arm and 130/100 mm Hg in the right arm). Laboratory results revealed normal C-reactive protein (CRP) and elevated creatinine; she performed angiography, which revealed bilateral renal artery stenosis.
Khan et al. [5]	A 33-year-old male presented to the emergency department for fever, myalgias, left arm numbness, and persistent hypertension. His CT aortogram showed multi-vessel narrowing, including renal arteries with an atrophic right kidney. After diagnosis, he was treated with oral corticosteroid and immunosuppressant therapy.
Pérez-García et al. [6]	A 22-year-old female was admitted to the emergency department with frontal headache and malaise; blood pressure was 197/92 mm Hg in the right arm and 199/95 mm Hg in the left arm. Computed tomography angiography (CTA) revealed concentric wall thickening of the thoracoabdominal aorta and stenosis of both renal arteries; fluorine-18 fluorodeoxyglucose positron emission tomography/computed tomography (18F-FDG-PET/CT) findings confirmed the inflammation of vascular walls supporting the diagnosis of Takayasu's arteritis.
Moya-Megías et al. [7]	A 46-year-old male presented with hypertensive emergency and subarachnoid hemorrhage due to aneurysmal rupture. The study of secondary causes of hypertension confirmed renal artery stenosis due to Takayasu's arteritis.
Rainer et al. [8]	A 30-year-old woman presented with uncontrolled hypertension 201/120 mmHg and renal failure. Laboratory results revealed increased serum creatinine of 9 mg/dl from a basal value of 0.9 mg/dl and oliguria. 18F-FDG-PET/CT showed increased FDG activity in both common carotid arteries. Angiography showed a complete occlusion of the bilateral renal arteries, suspicious for Takayasu's arteritis.
Ur Rahman et al. [9]	A 57-year-old male patient presented with a late diagnosis of Takayasu's arteritis arising as stroke in the left middle cerebral artery territory, left renal artery stenosis, and hypertension with differences of blood pressure between two arms due to predominant left subclavian artery stenosis.
Valente et al. [10]	A 65-year-old woman performed an angiological examination, complaining of intermittent claudication. Upon physical examination, the main findings were blood pressure not measurable in the left arm, bruit in her left-side subclavian and carotid arteries, and femoral pulse difference between the two legs. She performed a magnetic resonance angiography that showed occlusion of the left subclavian artery, the right common iliac artery, and also the superior mesenteric artery, critical stenosis at the origin of the celiac trunk, and moderate stenosis of the right renal artery. These findings were consistent with the diagnosis of Takayasu's arteritis.
Ozkok et al. [11]	A 23-year-old female patient with factor VII (FVII) deficiency was hospitalized with severe hypertension and renal failure. Brachial arterial pressures were 230/120 and 220/115 mm/Hg on the right and left arms, respectively. Renal artery Doppler ultrasonography revealed bilateral severe renal artery stenosis. Contrast-enhanced magnetic resonance imaging angiography revealed severe mural irregularities, contrast enhancement in the aorta and its branches, and long-segment stenosis starting in the abdominal aorta and extending into the proximal renal arteries.

In line with previous literature, our patient complained of neurological symptoms such as headache, dizziness, and gait instability and had evidence of impaired kidney function. However, our case presented a unique clinical finding that deserves to be highlighted: the diffuse cramps associated with the refractory and persistent hypokalemia as a consequence of severe hyperreninemic aldosteronism. In the diagnostic workup, imaging techniques recommended by EULAR are ultrasound, MRI, or positron emission tomography, which are particularly useful in the evaluation of the disease both in its active form and in response to treatment. In our case, the patient was initially evaluated with bedside ultrasound with the finding of diffuse hypoechoic thickening of the aorta (Figure 1) that led us to suspect a vasculitis even without laboratory findings consistent with active inflammation. So, based on our experience, initial point-of-care ultrasonography evaluation could provide extremely useful elements of suspicion of inflammatory vascular disease due to characteristic echographic findings. According to EULAR guidelines on the treatment of the active form of Takayasu's arteritis, we started therapy with high-dose steroids (1 mg/kg/day of methylprednisone) with early association of a tumor necrosis factor (TNF)-inhibitor due to the extension of the disease. Hypertension and hypokalemia management were difficult, and we obtained reasonable control of both conditions by combining amlodipine 10 mg/day, metoprolol 100 mg bid, chlortalidone 25 mg/day, and spironolactone 100 mg/day. We have to mention that surgical revascularization could be considered in cases of stenotic or occlusive lesions that are symptomatic or at risk of ischemic evolution, particularly in cases of uncontrolled arterial hypertension in renal artery stenosis, in the presence of aneurysmal dilatation at risk of dissection or severe aortic regurgitation and aortic coarctation. Fortunately, in our patient, we were able to reach reasonable blood pressure control with medical therapy; however, surgical revascularization has been scheduled to bypass the occlusion of the aorto-iliac bifurcation. In sum, Takayasu's arteritis could present with severe hypertension, so it has to be included in the differential diagnosis of renovascular hypertension. In the initial diagnostic workup, refractory hypokalemia due to hyperreninemic aldosteronism and ultrasonographic evidence of diffuse hypoechoic thickening of the aortic wall could be extremely useful elements to raise the clinical suspicion of systemic vasculitis.

## Conclusions

Takayasu's arteritis could rarely present itself with severe hypertension as the initial clinical manifestation of the disease. Maintaining high clinical suspicion and correct initial diagnostic approach is essential for prompt diagnosis and management. In renovascular hypertension, young age, female sex and the absence of major cardiovascular risk factors should be considered elements of clinical suspicion for rare diseases such as fibromuscular dysplasia or Takayasu's arteritis. Based on our experience, the initial diagnostic should include an evaluation of the renin-angiotensin-aldosterone system and bedside point-of-care ultrasonography. The presence of refractory hypokalemia due to hyperreninemic aldosteronism and ultrasonographic evidence of diffuse hypoechogenic thickening of the aortic wall could be considered extremely useful elements to raise the clinical suspicion of systemic vasculitis and guide doctors towards the most appropriate further testing.
